# Sodium zirconium cyclosilicate, hyperkalaemia, and spironolactone optimization in heart failure with reduced ejection fraction: The REALIZE‐K open‐label run‐in phase

**DOI:** 10.1002/ejhf.3787

**Published:** 2025-08-20

**Authors:** Mark C. Petrie, David Z.I. Cherney, Akshay S. Desai, Jeffrey M. Testani, Subodh Verma, Khaja Chinnakondepalli, David Dolling, Shachi Patel, Magnus Dahl, James M. Eudicone, Lovisa Friberg, Mario Ouwens, Murillo O. Antunes, Kim A. Connelly, Vagner Madrini, Luca Kuthi, Anuradha Lala, Miguel Lorenzo, Patrícia O. Guimarães, Marta Cobo Marcos, Béla Merkely, David Gonzales‐Calle, Julio Nuñez Villota, Iain Squire, Jan Václavík, Jerzy Wranicz, Mikhail N. Kosiborod

**Affiliations:** ^1^ School of Cardiovascular and Medical Sciences University of Glasgow Glasgow UK; ^2^ University Health Network and Mount Sinai Hospital and University of Toronto Toronto ON Canada; ^3^ Cardiovascular Division Brigham and Women's Hospital Boston MA USA; ^4^ Section of Cardiovascular Medicine Yale University Guilford CT USA; ^5^ Institute of Unity Health Toronto and University of Toronto Toronto ON Canada; ^6^ Saint Luke's Mid America Heart Institute and University of Missouri‐Kansas City Kansas City MO USA; ^7^ Fortrea Maidenhead UK; ^8^ BioPharmaceuticals Medical, AstraZeneca Gothenburg Sweden; ^9^ BioPharmaceuticals Medical (Evidence), AstraZeneca Wilmington DE USA; ^10^ BioPharmaceuticals Medical, AstraZeneca Cambridge UK; ^11^ Hospital Universitário São Francisco de Assis na Providência de Deus and Universidade São Francisco Bragança Paulista Brazil; ^12^ Keenan Research Centre for Biomedical Science, Unity Health Division Head, Cardiology, St. Michael's Hospital Toronto ON Canada; ^13^ Hospital Israelita Albert Einstein São Paulo Brazil; ^14^ Semmelweis University Budapest Hungary; ^15^ Mount Sinai Fuster Heart Hospital, Icahn School of Medicine New York NY USA; ^16^ Hospital Clínico Universitario de Valencia University of Valencia Valencia Spain; ^17^ Hospital Universitario Puerta de Hierro Majadahonda Madrid Spain; ^18^ Hospital Universitario de Salamanca Salamanca Spain; ^19^ Hospital Clinico Universitario de Valencia and University of Valencia Valencia Spain; ^20^ NIHR Cardiovascular Biomedical Research Unit, Glenfield Hospital Leicester UK; ^21^ University Hospital Ostrava and Faculty of Medicine University of Ostrava Ostrava Czech Republic; ^22^ Department of Electrocardiology Medical University of Lodz Łódź Poland; ^23^ Late‐Stage Development, Cardiovascular, Renal and Metabolic, BioPharmaceuticals R&D, AstraZeneca Boston MA USA

## Introduction

In heart failure with reduced ejection fraction (HFrEF), mineralocorticoid receptor antagonists (MRAs) reduce mortality and HF hospitalizations, and are one of the key cornerstones of guideline‐directed medical therapy.^1^, ^2^, ^3^, ^4^ Hyperkalaemia (or fear of hyperkalaemia) is a major reason for their underuse.^5^ In the randomized‐withdrawal phase of the REALIZE‐K trial, of patients with HFrEF and prevalent hyperkalaemia or at risk of hyperkalaemia, continued use of the potassium (K^+^) binder sodium zirconium cyclosilicate (SZC) led to large increases in the number of participants on optimal‐dose spironolactone with normokalaemia, and reduced the risk of hyperkalaemia and down‐titration/discontinuation of spironolactone compared with withdrawal to placebo.^6^ Prior to the placebo‐controlled, randomized‐withdrawal phase of REALIZE‐K, there was a run‐in phase in which SZC was used to manage hyperkalaemia and spironolactone dose was optimized.^7^

This analysis evaluated the efficacy of SZC in lowering serum (s)K^+^ and enabling SZC titration during the run‐in phase among those with prevalent hyperkalaemia on no or low‐dose (12.5 mg) spironolactone; and in those identified as at high risk of hyperkalaemia on no or low‐dose spironolactone, to evaluate the incidence of hyperkalaemia during spironolactone dose titration during the run‐in phase and use of SZC to lower sK^+^ and enable maintenance of spironolactone.

## Methods

This was a post‐hoc analysis of REALIZE‐K, which was a prospective phase 4, double‐blind, placebo‐controlled, randomized‐withdrawal trial evaluating the role of SZC in enabling MRA therapy in patients with HFrEF and hyperkalaemia.^7^ Patient eligibility criteria have been reported previously.^7^ Briefly, trial participants were required to have a left ventricular ejection fraction ≤40% and to be on a stable dose of angiotensin‐converting enzyme inhibitor, angiotensin receptor blocker, or angiotensin receptor–neprilysin inhibitor, as well as a beta‐blocker. Patients had to be either untreated with, or on a low dose of, MRA (<25 mg daily of spironolactone or eplerenone) because of either: prevalent hyperkalaemia (Cohort 1), defined as sK^+^ 5.1–5.9 mEq/L at screening and estimated glomerular filtration rate (eGFR) ≥30 ml/min/1.73 m^2^; or at high risk of hyperkalaemia (Cohort 2), defined as either documented history of hyperkalaemia (sK^+^ >5.0 mEq/L) in the previous 36 months and eGFR ≥30 ml/min/1.73 m^2^, or sK^+^ 4.5–5.0 mEq/L, and either eGFR 30–60 ml/min/1.73 m^2^ or aged >75 years.

Participants who fulfilled the eligibility criteria entered the open‐label run‐in phase. This analysis included all participants who entered the open‐label period and received at least one dose of SZC or spironolactone. In Cohort 1 (prevalent hyperkalaemia at screening), this was a 4‐week period during which patients were initiated on SZC on day 1 at a dose of 10 g three times daily for 48 h until sK^+^ was normalized (3.5–5.0 mEq/L). After sK^+^ normalization, SZC was down‐titrated or up‐titrated between 5 g every other day and 15 g daily to maintain sK^+^ 3.5–5.0 mEq/L as per protocol‐mandated instructions.^7^ Spironolactone was either initiated or up‐titrated to a target dose of 50 mg daily, as tolerated, per protocol‐mandated instructions.^7^

In Cohort 2 (high risk of hyperkalaemia), the open‐label run‐in phase could be extended up to 6 weeks. Spironolactone was initiated or up‐titrated on day 1 and was systematically up‐titrated to a target dose of 50 mg daily, as tolerated per protocol‐mandated instructions.^7^ Patients who experienced hyperkalaemia (sK^+^ >5.0 mEq/L) during the first 4 weeks of the run‐in phase were started on SZC 10 g three times daily for ≤48 h until sK^+^ normalized (3.5–5.0 mEq/L). Those who achieved normokalaemia were maintained on SZC 10 g daily, which could be down‐ or up‐titrated between 5 g every other day and 15 g daily to maintain normokalaemia per protocol‐mandated instructions.^7^

## Results

In the REALIZE‐K run‐in phase, 95 patients with prevalent hyperkalaemia (Cohort 1) and 271 at high risk of hyperkalaemia (Cohort 2) were enrolled. Ninety‐four in Cohort 1 and 268 in Cohort 2 received at least one dose of spironolactone. Ninety‐four in Cohort 1 and 147 in Cohort 2 received at least one dose of SZC.

**Figure 1:**
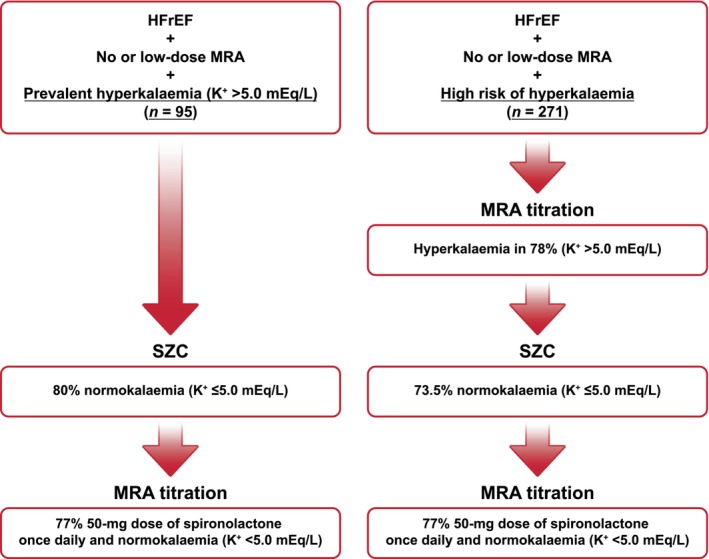
REALIZE‐K run‐in phase: chronic heart failure with reduced ejection fraction (HFrEF, left ventricular ejection fraction ≤40%), hyperkalaemia, sodium zirconium cyclosilicate (SZC), and titration of spironolactone. K^+^, potassium; MRA, mineralocorticoid receptor antagonist.

In Cohort 1, mean age was 70.5 years, mean K^+^ was 5.3 (± 0.4), 31.6% had type 2 diabetes, and mean eGFR was 56.4 ml/min/1.73 m^2^. SZC resulted in reduction of sK^+^ to ≤5.0 mEq/L within 48 h in 79.8% of patients (*Figure* *1*). In those who achieved normokalaemia, 77.0% achieved titration to 50 mg of spironolactone, 2.7% to 37.5 mg, 18.9% to 25 mg, and 1.4% to 12.5 mg over the subsequent 4 weeks. At the end of the open‐label phase, 11.6% of patients remained hyperkalaemic (sK^+^ >5.0 mEq/L) despite SZC titration; 1.1% had experienced an oedema‐related adverse event.

In Cohort 2, mean age was 69.9 years, mean K^+^ was 4.7 (± 0.4), 21.5% had type 2 diabetes, and mean eGFR was 59.3 ml/min/1.73 m^2^. Over 6 weeks, MRA dose titration to 50 mg once daily resulted in hyperkalaemia (sK^+^ >5.0 mEq/L) in 77.8% of patients (in 20.5%, MRA titration resulted in sK^+^ >5.5 mEq/L and in 1.8%, sK^+^ was >6.0 mEq/L). Those who did versus did not develop hyperkalaemia were more likely to have had atrial fibrillation, have a lower baseline eGFR, and a higher baseline sK^+^ and N‐terminal pro‐B‐type natriuretic peptide (*Table* *1*). In 73.5% of those who developed hyperkalaemia, SZC treatment resulted in resolution of hyperkalaemia (sK^+^ ≤5.0 mEq/L) within 48 h. Over the 6 weeks, 77.3% achieved titration to 50 mg of spironolactone, 2.3% to 37.5 mg, 19.5% to 25 mg, and 0.8% to 12.5 mg. At the end of the open‐label phase, 12.2% remained hyperkalaemic (sK^+^ >5.0 mEq/L) despite addition of SZC and 1.9% experienced an oedema‐related adverse event.

**Table 1:** Baseline characteristics of patients at high risk of hyperkalaemia who did or did not develop hyperkalaemia (K^+^ >5.0 mEq/L) during mineralocorticoid receptor antagonist titration

	Patients who developed hyperkalaemia (K^+^ >5.0 mEq/L) during MRA titration (*n* = 199)	Patients who did not develop hyperkalaemia (K^+^ >5.0 mEq/L) during MRA titration (*n* = 72)
Mean age, years	71.0	71.5
Female sex	47 (24)	20 (28)
Region		
Latin America	42 (21)	16 (22)
North America	42 (21)	8 (11)
Europe	115 (58)	48 (67)
Systolic blood pressure, mmHg	117	108
Atrial fibrillation/flutter	72 (36)	18 (25)
Type 2 diabetes	43 (22)	15 (21)
History of chronic kidney disease	83 (42)	20 (28)
eGFR screening, ml/min/1.73 m^2^	55.0	63.0
Serum K^+^ at screening, mEq/L	4.8	4.5
LVEF, %	34	32
NT‐proBNP, pg/ml	1247	1002
BMI, kg/m^2^	28	29
ICD or CRT‐D	63 (32)	14 (19)
CRT‐P	5 (2)	1 (1)
Previous heart failure hospitalization	99 (50)	34 (47)
NYHA functional class at screening		
I/II	173 (87)	56 (78)
III/IV	26 (13)	16 (22)
Medical therapy at screening		
ARNi	128 (64)	49 (68)
ACEi/ARB/ARNi	197 (99)	70 (97)
Beta‐blocker	189 (95)	71 (99)
SGLT2i	135 (68)	54 (75)
Diuretic agents	139 (70)	47 (65)
MRA (low dose at enrolment)	102 (51)	41 (57)

Values are reported as *n* (%) unless otherwise stated.

ACEi, angiotensin‐converting enzyme inhibitor; ARB, angiotensin receptor blocker; ARNi, angiotensin receptor–neprilysin inhibitor; BMI, body mass index; CRT‐D, cardiac resynchronization therapy with defibrillator; CRT‐P, cardiac resynchronization therapy with pacemaker; eGFR, estimated glomerular filtration rate; ICD, implantable cardioverter‐defibrillator; K^+^, potassium; LVEF, left ventricular ejection fraction; MRA, mineralocorticoid receptor antagonist; NT‐proBNP, N‐terminal pro‐B‐type natriuretic peptide; NYHA, New York Heart Association; SGLT2i, sodium–glucose co‐transporter 2 inhibitor.

## Discussion

In the run‐in phase of REALIZE‐K, SZC reduced sK^+^ to ≤5.0 mEq/L in 80% of those with prevalent hyperkalaemia within 48 h, and over the next 4 weeks enabled 80% of these patients to tolerate doses of spironolactone ≥25 mg. Among those defined as being at high risk of hyperkalaemia (based on prior history of hyperkalaemia or risk factors such as age or advanced chronic kidney disease), 78% developed hyperkalaemia during spironolactone up‐titration. Among these patients, SZC resulted in reduction of sK^+^ to ≤5.0 mEq/L in 73.5% of patients within 48 h of incident hyperkalaemia and enabled 87% to tolerate doses of spironolactone ≥25 mg.

There is ongoing uncertainty and debate about which definition of hyperkalaemia is optimal in terms of identifying risk of clinical events. This issue is of major relevance for many novel therapies in recently completed, ongoing and upcoming clinical trials to both treat and prevent heart failure. These novel therapies include non‐steroidal MRAs and aldosterone synthase inhibitors.

Hyperkalaemia occurs frequently in higher‐risk patients undergoing spironolactone up‐titration, suggesting that rechallenge with MRAs in this population should be undertaken with caution and careful laboratory surveillance. In most patients with prevalent hyperkalaemia or those at high risk of hyperkalaemia, use of SZC rapidly corrects sK^+^ to the normal range and allows initiation and titration of spironolactone to optimal doses without recurrent hyperkalaemia.
